# COVID-19 and Contact Tracing Apps: Ethical Challenges for a Social Experiment on a Global Scale

**DOI:** 10.1007/s11673-020-10016-9

**Published:** 2020-08-25

**Authors:** Federica Lucivero, Nina Hallowell, Stephanie Johnson, Barbara Prainsack, Gabrielle Samuel, Tamar Sharon

**Affiliations:** 1 Ethox and Wellcome Centre for Ethics and Humanities, Nuffield Department of Population Health, University of Oxford, Old Road Campus, Oxford, UK; 2 Department of Political Science, University of Vienna, Vienna, Austria; 3 Department of Global Health and Social Medicine, King’s College London, London, UK; 4 Department of Practical Philosophy & Interdisciplinary Hub for Security, Privacy and Data Governance, Radboud University, Nijmegen, The Netherlands

**Keywords:** Contact tracing apps, COVID-19, Transparency, Social experiment

## Abstract

Mobile applications are increasingly regarded as important tools for an integrated strategy of infection containment in post-lockdown societies around the globe. This paper discusses a number of questions that should be addressed when assessing the ethical challenges of mobile applications for digital contact-tracing of COVID-19: Which safeguards should be designed in the technology? Who should access data? What is a legitimate role for “Big Tech” companies in the development and implementation of these systems? How should cultural and behavioural issues be accounted for in the design of these apps? Should use of these apps be compulsory? What does transparency and ethical oversight mean in this context? We demonstrate that responses to these questions are complex and contingent and argue that if digital contract-tracing is used, then it should be clear that this is on a trial basis and its use should be subject to independent monitoring and evaluation.

## Introduction

The “Alipay Health Code” application (app) was developed by Ant Financial, a sister company of the technology company Alibaba and initially deployed in Hangzhou, China, during the COVID-19 outbreak. The Health Code algorithm matches actively collected data (self-reported symptoms, individual’s address, personal ID) and passively collected data such as GPS location, and assigns the individual a coloured code (Green, Amber, Red) which, when combined with scanned quick response (QR) codes, determines access to public areas, such as subways, malls, and markets (Mozur, Zhong, and Krolik 2020). A similar app, originally used during the Middle East respiratory syndrome (MERS) coronavirus epidemics, has been deployed in South Korea. Another one, “Trace Together,” has been launched by the Singapore government as a tool to support and supplement manual contact-tracing utilizing Bluetooth wireless technology.

Several countries have shown interest in digital contact-tracing apps since mid-March 2020, and in Europe there has been widespread media coverage of academic and corporate developments and government agencies’ statements and actions concerning their adoption. The general understanding of digital contact-tracing apps is as follows: people download an app that communicates with other devices in the proximity, typically via Bluetooth; Person A develops symptoms/tests positive and enters this information into the app; people who have been within a certain predefined proximity of Person A’s phone for a specified timeframe are notified by text message and advised to self-isolate. Despite the fact that the European Commission has recommended a pan-European approach to the adoption of digital contact-tracing apps and outlined principles as well as practical measures for inter-state and inter-agency collaborations (European Commission 2020), controversies have arisen among and within countries about the adoption of these apps and different, and often incompatible, systems have been developed.

There is widespread agreement that digital technologies may prove useful tools when exiting national lockdowns in the current COVID-19 crisis. At the same time, there is increasing concern that the temporary restrictions that digital surveillance entails could lead to a more permanent suspension of rights and liberties and could have some unintended consequences. After some brief notes on the exceptionality of digital contact-tracing in the context of public health surveillance, this paper explores some crucial questions that we believe need to be addressed when assessing the ethical challenges raised by the deployment of contact-tracing apps in the containment of this pandemic.

## Surveillance and Public Health

Patient data is routinely collected and curated for the purpose of disease surveillance, for example, in cancer and notifiable disease registries. Typically, traditional epidemiological surveillance has been based on data collected by public health agencies through health personnel in hospitals, doctors’ offices, and out in the field (Salathé et al. 2012). More recently, novel data sources have emerged where data are collected directly from individuals through the digital traces they leave (Eysenbach 2009). Data from search engines can now provide early warning of respiratory illnesses in local communities, data from social networking sites can provide early warning of vaccine refusal, and tracking population movements with mobile phone network data has improved responses to disasters and outbreaks (Bengtsson et al. 2011). One of the key advantages of digital surveillance using contact-tracing apps, apart from the increasingly large data volumes, is that they are contextual and networked (Salathé and Khandelwal 2011). This allows the study of individuals and groups in context and the study of person-to-person spread of disease as well as behaviours that may influence infection risk (Salathé et al. 2012). These advantages also raise important questions about the extent of the legitimacy of such highly individual and contextualized surveillance methods in emergency situations.

In the context of infectious disease control, contact-tracing has traditionally been an important means of disease control: identifying infected individuals and informing the people they have been in contact with that they are at risk, through a meticulous process of retracing where and with whom an infected individual has been in proximity. Automated contact-tracing offers several advantages over traditional contact-tracing in the case of the COVID-19 pandemic. First, it seeks to automate a labour-intense practice in a situation where there is a scarcity of human contact tracers. Moreover, it may offer more accuracy where human memories are fallible—particularly in the case of COVID-19, where infection can be asymptomatic for up to two weeks (Kimball et al. 2020). At the same time, it raises questions about the possibility of trusting algorithms with crucial public health decisions that may have critical consequences for individuals.

To better understand digital contact-tracing apps, we need to contextualize them within the ongoing trends in digital data use and automation of public health practices. We also need to deal with important questions that have been raised about the extent of the legitimacy of such highly granular surveillance methods and automated mechanisms, as well as the concern that if implemented in an emergency they may remain with us for a long time (Harari 2020). Despite the urgency of decision-making in public health emergencies, a rigorous ongoing evaluation of the legitimacy of crisis measures is paramount (Nuffield Council of Bioethics 2020). In the following, we highlight questions that are crucial to address in order to justify a legitimate and ethical use of these apps.

## Which Safeguards Should be Designed into the Technology?

Technologies can be designed in such a way that values, ethical norms, and legal principles are built in (Hildebrandt and Tielemans 2013). As such, design decisions can act as powerful limitations for possible excesses in times of crisis, and getting the relevant technical design of contact-tracing apps right is key. Whether these apps will collect geolocation data via GPS, communicate via Wi-Fi or Bluetooth signals, whether the data will be stored locally on users’ devices or exported to centralized databases run by governments or health authorities, whether these data can also be used for research, whether users have any control over who can access their data, and whether data are automatically deleted once the pandemic is over: these are all decisions that translate ethical and legal concerns including consent, purpose limitation, data minimization, and data protection into technical design. They require careful deliberation that includes the expertise and voices not just of technology developers but also privacy and human rights advocates, ethicists, and affected groups, to consider the trade-offs of different technical approaches. Importantly, value-based decisions are not solely concerned with the app as a technical object but also with the design of the institutional, legal, and organizational measures that will be created around these apps.

## Who Accesses the Data?

In South Korea, contact-tracing app data on the movement of individuals were made publicly available on a government website, however in other countries it is often less clear which authorities and other institutions can access the data. Initial debates about the legitimacy of these apps have mostly focused on issues of privacy and surveillance as commentators emphasized the differences between a Korean and Chinese acceptance of state surveillance and a European scepticism towards this practice. The issue of who should access the data produced by these apps has been at the core of the controversy around the desirability of a “centralized” versus a “decentralized” or “localized” approach. The first method collects data (about COVID-19 symptoms, positive test, and contacts) in a central database that are subsequently accessible for epidemiological and public health purposes. The decentralized approach notifies at-risk contacts automatically but does not retain health or contact data and therefore these data are not accessible for research or other purposes (Troncoso et al. 2020). A number of European authorities and privacy advocacy groups have emphasized that accessibility to a central server comes with risks for individual privacy and autonomy, as well as the possibility of state surveillance and third-party data breaches. Lack of data accessibility prevents these risks but may jeopardize other important and socially valuable functionalities that these apps may have, such as the collection of epidemiological data for research or public health service planning.

## What Should the Role of “Big Tech” Be?

In response to the crisis, large information technology companies such as Alphabet, Microsoft, Apple, Facebook, and Palantir in the West, and Alibaba and Baidu in China, have been invited to contribute to pandemic response strategies in various ways. These include the development of disease surveillance tools, the setting up of testing sites, the use of artificial intelligence (AI) for diagnostics, and the funding of COVID-19 related research. In a rare collaboration, Apple and Google have also provided the architecture for exposure notification apps (Application Programming Interface or API) that enables the communication between devices that have downloaded the contact-tracing app. This system has been introduced by these technology giants as a way of preserving users’ privacy as it maintains data on their devices rather than transmitting to central servers. But while the technical expertise and financial resources that these companies bring to the table may be welcome contributions in times of a global public health crisis, we should be wary of the costs these contributions carry for society further down the line (Sharon 2018; Prainsack 2020). Namely, what kind of dependencies are created on these actors, who are already so powerful in other domains, and to what extent are they taking over functions of public sector actors in the provision of public services? Here too, careful deliberation about the long-term trade-offs involved in immediate pandemic response strategies is required.

## Efficacy and Use: Enough Evidence?

Questions remain regarding how digital surveillance apps designed for pandemic containment will work in practice. This is because app efficacy is premised on a set of assumptions about human behaviour—for example, assumptions that relate to the fact that 60 per cent of the population will download the app and use it appropriately. Reaching this number is not an easy task: reported download numbers in some countries are very low (only 12 per cent in Singapore). Assumptions about higher levels of app acceptance and use can fail to recognize problems such as a large proportion of the population not having mobile phones able to support these apps and these populations happening to be the more vulnerable ones. This is an example of how the assumptions about human behaviour that drive the development and implementation of contact-tracing apps may foster social inequalities if not addressed promptly. It should also be noted that many variables, including a lack of trust in the system, may affect uptake and adherence to the app’s recommendations to self-isolate. As it has been pointed out by behavioural scientists (West et al. 2020), issues of acceptability, affordability, effectiveness, practicability, spill-over, and equity, are paramount to discussions of the human and social components of these technological responses and (as we add) anticipation of their ethical challenges.

## Compulsory or Voluntary?

Lack of compliance with lockdown measures in some countries has required stricter enforcement using criminal and pecuniary measures. Despite this, it is suggested that the use of tracking apps should be an individual choice.^1^ This more libertarian approach to the adoption of tracking apps is not straightforward. First, we can ask to what extent the responsibility for a public health matter should be placed on individuals and what this means in terms of accountability. Furthermore, one can imagine that in some countries, as in China, individuals could be required to use contact tracing apps if they wish to participate in certain activities (e.g. enter public/private spaces) or use public transport (Parker et al. 2020). These restrictions would make the app *de facto* compulsory if individuals are to remain functioning members of society and would result in discrimination against those who either refuse to use it or do not have smartphones (the public provision of rental mobile phones would be a remedy for the latter but not for the former). It could be argued that making the use of tracking apps compulsory is more transparent, and therefore, may be more morally acceptable, than an in-principle voluntary (but *de facto* constraining) approach. However, advocating compulsory adoption to overcome the problem of free riders and avoid *de facto* restrictions cannot overcome the fact that certain groups within society may not be able to access this technology and therefore, if apps are needed to access certain activities, compulsory adoption will result in the creation of a group of people whose freedoms are inequitably curtailed.

## Transparency, Oversight, and Accountability

At the time of writing, the questions explored here are currently dividing commentators on the acceptability of contact-tracing apps, while decision-makers are (largely) keeping silent. But transparency is crucial to maintain legitimacy. It is a means to accountability and is especially required when responses to crises involve exceptional measures that affect individuals’ liberty. In the case of contract-tracing apps**,** citizens deserve clarity about many aspects of their implementation: the purpose of data collection, the types of data collected, the parties who have access to them, the extent, modalities, and timeline for data deletion, the algorithms and data training sets that will automate processes and influence their daily lives. At present only technical experts are involved in their oversight and assessment,^2^ through governance mechanisms that are often unclear. Given their potential to threaten privacy and individual liberty and to foster inequalities, robust oversight of the deployment of these surveillance technologies, which involves users and civil society groups, is urgently needed. Finally, the uncertainties, difficulties and knowledge gaps related to these apps should also be disclosed. It is problematic for politicians or policymakers to portray contact-tracing apps as an easy solution to ease our way out of lockdown and mitigate new waves of infection. Conditions of respect for people’s privacy, protection of their data, limiting surveillance to the minimum necessary to overcome the current crisis, addressing potential issues of discrimination, as well as conditions for the involvement of powerful private actors in this crisis that adhere to values of democratic governance, need to be rigorously met for national governments to approve of their use.

After these conditions are met, it seems legitimate to treat and evaluate these apps for what they are: one of the many experimental solutions proposed to manage this pandemic. Acknowledging the experimental nature of these technologies means we need to follow a more cautious approach to their adoption and be prepared to monitor and independently evaluate their efficacy and utility. This would mean ensuring that, like other experiments (e.g. clinical trials), there are oversight mechanisms in place which: monitor the societal consequences of the use of apps (for example, the creation of social inequalities through digital exclusion), protect citizens who volunteer to participate in this experiment, and outline clear mechanisms for accountability (delineating who is legally responsible if something goes wrong). This means that we need to develop a transparent organizational and governance infrastructure around these apps. Finally, if all these conditions are put in place and it appears that these apps do not work as expected, or their harmful side-effects outweigh the promised benefits, then their use must be challenged and terminated.

## Footnotes

1 As emphasized by Germany's Justice Minister Christine Lambrecht. See Martin, M. 2020. German minister says tracking apps to tackle coronavirus must be voluntary. *Reuters*, March 31, 2020. https://www.reuters.com/article/us-health-coronavirus-germany-app/german-minister-says-tracking-apps-to-tackle-coronavirus-must-be-voluntary-idUSKBN21I0KM. Accessed July 22, 2020.

2 In Italy, for example, a multidisciplinary taskforce of seventy-four experts were appointed to evaluate the more than three hundred proposals of tracking apps that have been submitted to the government. See statement from the Italian Government Minister for Innovation and Digitization, “Nasce la task force italiana per l’utilizzo dei dati contro l’emergenza Covid-19” March 31, 2020. https://innovazione.gov.it/nasce-la-task-force-italiana-per-l-utilizzo-dei-dati-contro-l-emergenza-covid-19/. Accessed July 22, 2020.

## References

[CR1] Bengtsson L, Lu X, Thorson A, Garfield R, von Schreeb J (2011). Improved response to disasters and outbreaks by tracking population movements with mobile phone network data: A post-earthquake geospatial study in Haiti. PLoS Medicine.

[CR2] Eysenbach G (2009). Infodemiology and infoveillance: Framework for an emerging set of public health informatics methods to analyze search, communication and publication behavior on the Internet. Journal of Medical Internet Research.

[CR3] European Commission. 2020. Commission Recommendation (EU) 2020/518 of 8 April 2020 on a common Union toolbox for the use of technology and data to combat and exit from the COVID19 crisis, in particular concerning mobile applications and the use of anonymised mobility data. *Official Journal of the European Union* (L 114/7), April 14. https://eur-lex.europa.eu/legal-content/EN/TXT/PDF/?uri=CELEX:32020H0518&from=EN. Accessed July 22, 2020.

[CR4] Harari, Y.N. 2020. Yuval Noah Harari: The world after coronavirus. *Financial Times*, March 20. https://www.ft.com/content/19d90308-6858-11ea-a3c9-1fe6fedcca75. Accessed July 22, 2020.

[CR5] Hildebrandt M, Tielemans L (2013). Data protection by design and technology neutral law. Computer Law and Security Review.

[CR6] Kimball, A., K.M Hatfield, M. Arons, et al. 2020. Asymptomatic and presymptomatic SARS-CoV-2 infections in residents of a long-term care skilled nursing facility—King County, Washington, March 2020. *Morbidity and Mortality Weekly Report (MMWR)* 69: 377–381. 10.15585/mmwr.mm6913e1 PMC7119514 32240128

[CR7] Mozur, P. R. Zhong, and A. Krolik. 2020. In coronavirus fight, China gives citizens a color code, with red flags. *The New York Times*, March 1. https://www.nytimes.com/2020/03/01/business/china-coronavirus-surveillance.html. Accessed July 22, 2020.

[CR8] Nuffield Council of Bioethics. 2020. Rapid policy briefing: Ethical considerations in responding to the COVID-19 pandemic, March 17. https://www.nuffieldbioethics.org/assets/pdfs/Ethical-considerations-in-responding-to-the-COVID-19-pandemic.pdf. Accessed July 22, 2020.

[CR9] Parker, M., C. Fraser, L. Abeler-Dorner, and D. Bonsall. 2020. The ethics of instantaneous contract tracing using mobile phone apps in the control of COVID-19 pandemics. https://github.com/BDI-pathogens/covid-19_instant_tracing/blob/master/ The ethics of instantaneous contract tracing using mobile phone apps in the control of pandemics.pdf. Accessed July 22, 2020. 10.1136/medethics-2020-106314 PMC7231546 32366705

[CR10] Prainsack, B. 2020. The political economy of digital data: Introduction to the special issue. *Policy Studies*. ePub ahead of print, February 10. 10.1080/01442872.2020.1723519.

[CR11] Salathe M, Khandelwal S (2011). Assessing vaccination sentiments with online social media: Implications for infectious disease dynamics and control. PLoS Computational Biology.

[CR12] Salathé M, Bengtsson L, Bodnar TJ (2012). Digital epidemiology. PLoS Computational Biology.

[CR13] Sharon, T. 2018. When digital health meets digital capitalism, how many common goods are at stake? *Big Data and Society* 5(2): 10.1177/2053951718819032.

[CR14] Troncoso, C., M. Payer, J-P. Hubaux, et al. 2020. Decentralized privacy—Preserving proximity tracing. https://github.com/DP-3T/documents/blob/master/DP3T White Paper.pdf. Accessed July 22, 2020.

